# Bioinformatics analysis identifies potential ferroptosis key genes in the pathogenesis of diabetic peripheral neuropathy

**DOI:** 10.3389/fendo.2023.1048856

**Published:** 2023-05-12

**Authors:** Ming Tian, Jin Yong Zhi, Fan Pan, Yong Zhu Chen, Ai Zhong Wang, Hui Ying Jia, Rong Huang, Wen Hui Zhong

**Affiliations:** ^1^ Burns Department, Shanghai Jiao Tong University Affiliated Ruijin Hospital, Shanghai, China; ^2^ Department of General Surgery, Putuo Hospital Shanghai University of Traditional Chinese Medicine, Shanghai, China; ^3^ Department of Anesthesiology, Shanghai Jiao Tong University Affiliated Sixth People's Hospital, Shanghai, China; ^4^ Department of Endocrine and Metabolic Diseases, Shanghai Institute of Endocrine and Metabolic Diseases, Ruijin Hospital, Shanghai Jiao Tong University School of Medicine, Shanghai, China; ^5^ Shanghai National Clinical Research Center for Metabolic Diseases, Key Laboratory for Endocrine and Metabolic Diseases of the National Health Commission of the People's Republic (PR) China, Shanghai, China; ^6^ Shanghai Key Laboratory for Endocrine Tumor, State Key Laboratory of Medical Genomics, Ruijin Hospital, Shanghai Jiao Tong University School of Medicine, Shanghai, China

**Keywords:** diabetic peripheral neuropathy, ferroptosis, bioinformatics, oxidative stress, immune-environment

## Abstract

**Background:**

Diabetic peripheral neuropathy (DPN) is a serious complication in Diabetes Mellitus (DM) patients and the underlying mechanism is yet unclear. Ferroptosis has been recently intensively researched as a key process in the pathogenesis of diabetes but there yet has been no related bioinformatics-based studies in the context of DPN

**Methods:**

We used data mining and data analysis techniques to screen differentially expressed genes (DEGs) and immune cell content in patients with DPN, DM patients and healthy participants (dataset GSE95849). These DEGs were then intersected with the ferroptosis dataset (FerrDb) to obtain ferroptosis DEGs and the associated key molecules and miRNAs interactions were predicted.

**Results:**

A total of 33 ferroptosis DEGs were obtained. Functional pathway enrichment analysis revealed 127 significantly related biological processes, 10 cellular components, 3 molecular functions and 30 KEGG signal pathways. The biological processes that were significantly enriched were in response to extracellular stimulus and oxidative stress. Key modules constructed by the protein–protein interaction network analysis led to the confirmation of the following genes of interest: DCAF7, GABARAPL1, ACSL4, SESN2 and RB1. Further miRNA interaction prediction revealed the possible involvement of miRNAs such as miR108b-8p, miR34a-5p, mir15b-5p, miR-5838-5p, miR-192-5p, miR-222-3p and miR-23c. Immune-environment content of samples between DM and DPN patients revealed significant difference in the levels of endothelial cells and fibroblasts, which further speculates their possible involvement in the pathogenesis of DPN.

**Conclusion:**

Our findings could provide insight for investigations about the role of ferroptosis in the development of DPN.

## 1 Introduction

Diabetic peripheral neuropathy (DPN) is one of the most common complication of patients with Diabetes Mellitus (DM) and is characterized by the presence of symptoms and/or signs of peripheral nerve dysfunction (1, 2). The mechanisms underlying the natural course of DPN are yet unclear but the recent resurgence of interest in the possible role abnormalities in programmed cell-death (PCD) signaling cascades has led to new leads in investigation (3, 4). Non-apoptotic regulated cell death has been reported in the pathogenesis of various neurological diseases and first defined in 2012, ferroptosis is characterized by iron-dependent accumulation of lipid peroxides which leads to oxidative stress and cell death and has been linked with the pathogenesis of DM complications, such as myocardial ischemia, epithelial dysfunction and nerve function degeneration (5, 6).

Recently, it was shown that due to the disorder of cellular metabolic pathways associated with iron metabolism, redox homeostasis and mitochondrial activity, Ferroptosis is involved in the evolution of DM complications (7–9). In a Streptozotocin (STZ)-induced mouse DM model *in vivo*, Wang et al. discovered significant changes in markers related to ferroptosis in DPN mice, such as ACSL4 which marks lipid peroxide accumulation and GPX4 which marks regulation of redox homeostasis (10). It is believed that the imbalance of redox homeostasis leads to the accumulation of reactive oxygen species (ROS) that promote ferroptosis (11, 12). Previous reports have speculated the possible association of the enhancement of the HIF-1α/HO-1 pathway, the regulation of the NRF2 downstream targets by HMGB1 and salusin-β, and the regulation of prfdx6 gene by Sp-1 gene with the induction of ferroptosis, eventually leading to the incidence of DNP (13–16). Nevertheless, the underlying complete molecular mechanism and pathophysiological process still require further study.

At present, there have been no bioinformatics-based studies on the mechanism of ferroptosis genes in the development of DPN. Therefore, we used data mining and data analysis techniques to screen differentially expressed genes (DEGs) in patients with DPN, DM patients and healthy participants. These DEGs were then intersected with the ferroptosis dataset to obtain ferroptosis DEGs. Moreover, to identify crucial biomarkers and establish the pathogenesis of DPN at the molecular level, we investigated key proteins and predicted possible miRNAs interactions that may play principal roles in DPN. Furthermore, by comparing the immune-microenvironment content of DM and DPN pathology samples, we investigated the possibility of target cells, which might prompt further prevention or treatment options. Our results will help to understand the role of ferroptosis in the incidence of DPN after and provide new thoughts for therapeutic interventions in the management of DM and its complications.

## 2 Materials and methods

### 2.1 Data source and microarray data

The clinical data of DPN patients, DM patients and healthy participants was obtained from NCBI Gene Expression Omnibus (GEO) (dataset GSE95849) (17). At the same time, the probe annotation information of the corresponding platform (PL22448 Phalanx Human lncRNA OneArray v1_mRNA) was downloaded, the probes were converted into gene symbols, and the probes that were not aligned to gene symbols were removed. These datasets were used for further analysis and mining. For multiple probes mapped to the same gene symbol, the average value of the probe was calculated as the expression level of the gene. See the attachment for the original data. Since the clinical data were obtained from public databases, so the consent of patients and ethics committee approval were unnecessary.

### 2.2 Differential expression analysis

To observe the molecular mechanism of disease occurrence, limma package Version3.10.3 in r3.6.1 language was used (http://www.bioconductor.org/packages/2.9/bioc/html/limma.html) (18). The linear regression and empirical Bayes methods provided were used to analyze the differential expression genes (DEGs) of DPN vs DM, and the corresponding P value and log2FC. In addition, the Benjamini & Hochberg method was used for multiple test correction to obtain the corrected p value, i.e. adjusted P-value. We evaluated it from the two levels of difference multiple and significance. The difference expression threshold was set as follows: a | log2 (fold-change) |>1 and adjusted P-value<0.05. Pheatmap in r3.6.1 (https://cran.r-project.org/web/packages/pheatmap/index.html) Version 1.0.8 (19) was used to show the expression of top100 DEGs (50 up and 50 down-regulated genes).

### 2.3 Identification of ferroptosis DEGs

We also obtained a dataset (FerrDb) that included 265 genes from the Ferroptosis Database (FerrDb; zhounan.org) and intersected it with GSE95849 to identify ferroptosis DEGs. The different iron death genes were obtained and used for subsequent analysis. The online tool Venny2.1 was employed to generate a Venn diagram of DEGs.

### 2.4 Functional enrichment analysis of ferroptosis DEGs

For the ferroptosis DEGs genes obtained in the previous step, the R package ‘clusterprofiler’ was used (http://bioconductor.org/packages/release/bioc/html/clusterProfiler.html, Version 4.0.5) (20). Kyoto Encyclopedia of Genes and Genomes (KEGG) analysis was obtained from the GSEA of WebGestalt. Gene ontology, referred to as GO, is an international standardized gene function classification system. It provides a set of dynamically updated standard vocabularies to comprehensively describe the properties of genes and gene products in organisms. GO function and KEGG pathway enrichment analysis were performed, focusing on the KEGG pathway analysis and KEGG enrichment analysis to find the pathways where the target gene is involved and analyze the significance of each pathway. The most significant TOP10 then obtained.

### 2.5 Ferroptosis DEGs protein–protein interaction network analysis

To predict protein–protein interactions (PPIs), STRING, (https://string-db.org/) an online database which can retrieve the interaction between a group of proteins, was utilized in the PPI network analysis. The PPI network was built and visualized by Galluvial package (version 0.12.3, https://CRAN.R-project.org/package=ggalluvial).

### 2.6 Prediction of miRNA-mRNA relationship

In addition to the abovementioned analytical tools, we also used the online database mirtarbase (http://mirtarbase.mbc.nctu.edu.tw/index.html) to predict targeted pivotal miRNAs and build the gene–miRNA interaction networks using Cytoscape v3.6.1 software (21).

### 2.7 Immune microenvironment analysis

For the analysis of the immune microenvironment, the following two algorithms are used to evaluate the immune microenvironment status of the samples, and Wilcoxon was used to test the difference of infiltration in DPN vs diabetes:

(1) MCPcounter is an absolute counting method. The R package MCPcounter (http://riskscore://github.com/ebecht/MCPcounter) (22) was used and based on the expression matrix of all mRNAs, the relative infiltration abundance of 10 immune cells in each sample was estimated.
(2) Using the ESTIMATE (23) algorithm, the stromal score and immune score of each sample were estimated from the expression data to represent the presence of stroma and immune cells.

## 3 Results

### 3.1 Acquisition of differentially expressed genes

Based on the expression matrix of geo data, limma package was used to analyze the differential expression between the comparison groups of DPN vs DM patients, and 1447 DEGs meeting the screening criteria were screened.

**Figure 1:**
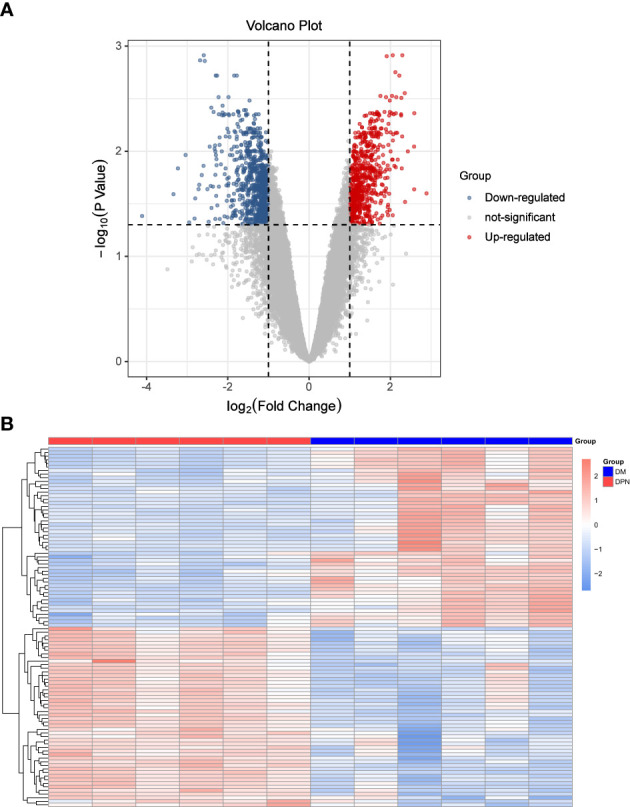
Differential Expression Analysis. **(A)** Volcano Plot: DEGs expression. **(B)** Heat map: Expression of top100 DEGs (50 up and 50 down-regulated genes).

See **Figures 1A** Volcano map and **1B** Heat map for details.

### 3.2 Identification of ferroptosis DEGs

**Figure 2:**
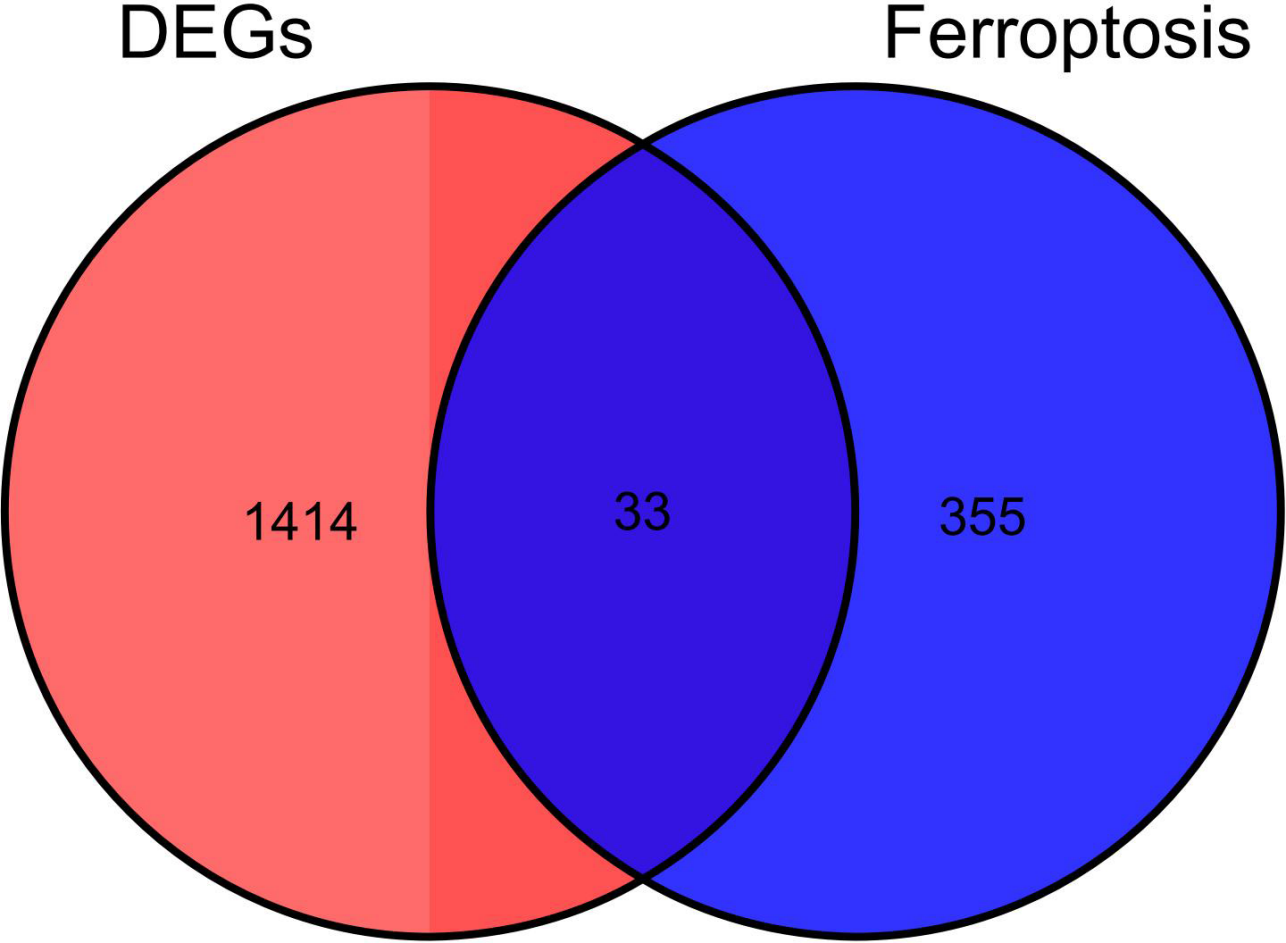
Identification of Ferroptosis DEGs: Venn diagram representing DEGs expression.

After the DEGs were obtained, they were intersected with 388 ferroptosis genes from the Ferroptosis Database (FerrDb): We found altogether 33 ferroptosis DEGs (**Table 1**), of which 14 genes were found to be up-regulated and the 19other down-regulated. The Venn diagram of the DEGs is shown in **Figure 2**.

**Table 1:** Characteristics of the 33 ferroptosis DEGs between DPN and DM patient database.

DEGs| Ferroptosis	log2FC	AveExpr	t	P Value	Adjusted P-value	B	Differential Expression
DCAF7	2.352	8.368	7.384	0	0.003	5.275	Up-Regulated
RB1	2.034	9.02	6.487	0	0.004	3.847	Up-Regulated
ELAVL1	1.16	8.574	5.019	0	0.01	1.242	Up-Regulated
ANO6	1.9	8.813	4.535	0	0.015	0.318	Up-Regulated
SCP2	1.612	10.164	4.519	0	0.015	0.288	Up-Regulated
CYBB	1.307	12.955	4.364	0.001	0.017	-0.014	Up-Regulated
CD44	1.426	11.287	3.771	0.002	0.029	-1.18	Up-Regulated
LAMP2	1.548	7.743	3.739	0.002	0.03	-1.243	Up-Regulated
ACSL4	1.37	10.078	3.686	0.002	0.032	-1.347	Up-Regulated
ACSL3	1.11	7.441	3.639	0.002	0.033	-1.439	Up-Regulated
IDH1	1.448	9.549	3.613	0.002	0.034	-1.491	Up-Regulated
FH	1.127	8.644	3.344	0.004	0.043	-2.02	Up-Regulated
EIF2AK4	1.063	7.79	3.27	0.005	0.047	-2.166	Up-Regulated
DPP4	1.189	8.316	3.243	0.005	0.048	-2.217	Up-Regulated
TFAP2C	-2.684	8.433	-8.43	0	0.001	6.783	Down-Regulated
HAMP	-1.45	8.884	-5.855	0	0.006	2.765	Down-Regulated
NNMT	-1.814	5.223	-5.694	0	0.007	2.479	Down-Regulated
MT3	-1.615	13.745	-5.544	0	0.007	2.209	Down-Regulated
HERPUD1	-1.459	13.338	-4.73	0	0.013	0.693	Down-Regulated
GRIA3	-1.431	4.066	-4.237	0.001	0.019	-0.261	Down-Regulated
HIC1	-2.703	8.643	-4.138	0.001	0.021	-0.456	Down-Regulated
MAP1LC3A	-2.423	9.884	-4.099	0.001	0.021	-0.532	Down-Regulated
ATF4	-1.093	13.579	-4.062	0.001	0.022	-0.606	Down-Regulated
HSPB1	-1.091	14.418	-4.057	0.001	0.022	-0.615	Down-Regulated
RELA	-1.317	13.197	-4.038	0.001	0.023	-0.652	Down-Regulated
BRD2	-1.224	14.401	-3.996	0.001	0.023	-0.736	Down-Regulated
GABARAPL1	-1.926	9.246	-3.625	0.002	0.034	-1.467	Down-Regulated
MAPK3	-1.569	11.43	-3.594	0.003	0.035	-1.529	Down-Regulated
PLIN2	-1.87	11.807	-3.492	0.003	0.038	-1.729	Down-Regulated
SLC7A5	-1.42	8.754	-3.458	0.003	0.039	-1.796	Down-Regulated
YY1AP1	-1.213	12.55	-3.403	0.004	0.041	-1.905	Down-Regulated
SESN2	-2.082	10.081	-3.398	0.004	0.042	-1.915	Down-Regulated
LONP1	-1.119	11.639	-3.233	0.005	0.048	-2.237	Down-Regulated

### 3.3 Functional pathway enrichment analysis

**Figure 3:**
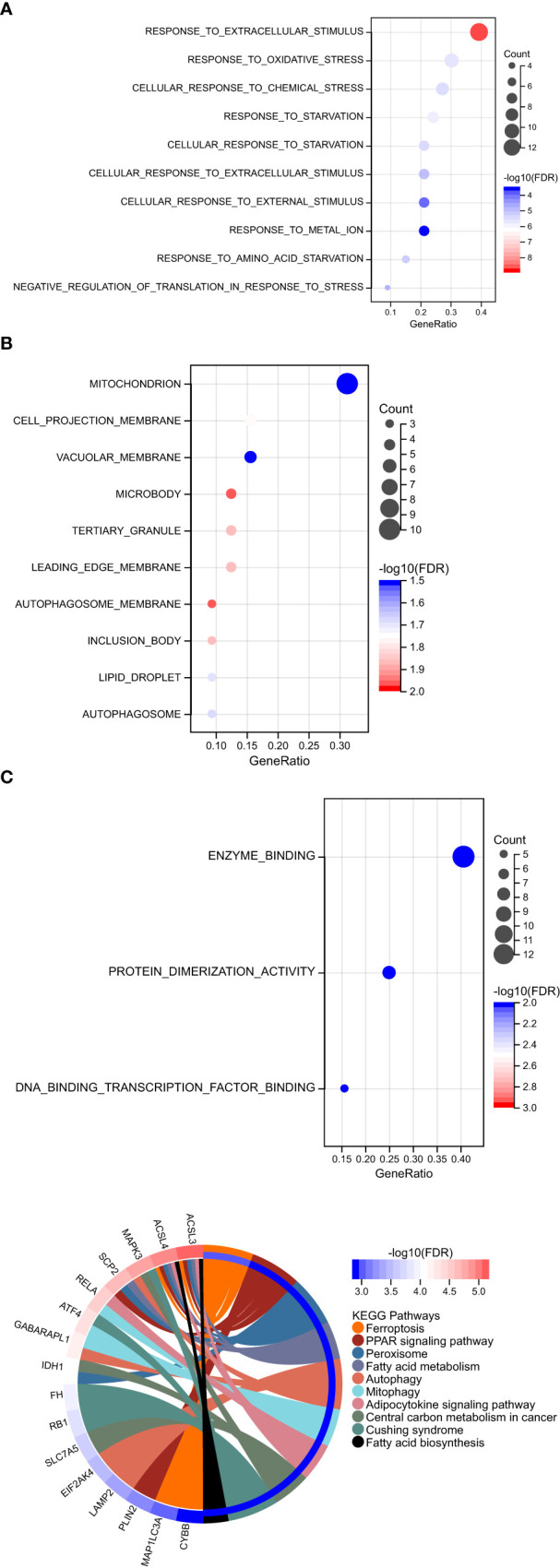
Functional pathway enrichment analysis. **(A)** 127 significantly related biological processes (BP) were involved. **(B)** 10 cellular components were involved. **(C)** 30 KEGG signal pathways were involved.

GO function and KEGG signal pathway enrichment analyses were performed on the 33 ferroptosis DEGs obtained in the previous step to explore the functional term involved by key genes. Gene Ontology: the enrichment results are shown in **Figure 3**. With adjusted P-value < 0.05 and count > 2 as thresholds, 127 significantly related biological processes (BP) (**Supplementary Table 1**; **Figure 3A**), 10 cellular components (CC) (**Table 2**; **Figure 3B**), 3 molecular functions (MF) (**Table 3**; **Figure 3C**) and 30 KEGG signal pathways (**Table 4**; **Figure 3D**) were analyzed. The results showed that the biological processes that were significantly enriched were in response to extracellular stimulus.

**Table 2:** Cellular components enrichment analysis.

Description	GeneRatio	BgRatio	P value	adjusted P Value	Q value	geneID	Count
MICROBODY	4/32	136/14140	0.00023888	0.01057	0.006789	SCP2/ACSL4/ACSL3/IDH1	4
AUTOPHAGOSOME_MEMBRANE	3/32	40/14140	9.83E-05	0.01057	0.006789	LAMP2/MAP1LC3A/GABARAPL1	3
TERTIARY_GRANULE	4/32	164/14140	0.00048692	0.013539	0.008696	ANO6/CYBB/LAMP2/IDH1	4
LEADING_EDGE_MEMBRANE	4/32	173/14140	0.00059555	0.013539	0.008696	CD44/DPP4/ATF4/GABARAPL1	4
INCLUSION_BODY	3/32	74/14140	0.00061195	0.013539	0.008696	MT3/HERPUD1/ATF4	3
CELL_PROJECTION_MEMBRANE	5/32	337/14140	0.00088531	0.017411	0.011183	CD44/DPP4/ATF4/GABARAPL1/SLC7A5	5
LIPID_DROPLET	3/32	95/14140	0.00126525	0.020359	0.013076	ACSL4/ACSL3/PLIN2	3
AUTOPHAGOSOME	3/32	99/14140	0.00142499	0.021019	0.0135	LAMP2/MAP1LC3A/GABARAPL1	3
MITOCHONDRION	10/32	1627/14140	0.00234766	0.031964	0.02053	SCP2/ACSL4/ACSL3/IDH1/FH/TFAP2C/GABARAPL1/MAPK3/SESN2/LONP1	10
VACUOLAR_MEMBRANE	5/32	431/14140	0.00262344	0.033168	0.021303	LAMP2/DPP4/MAP1LC3A/GABARAPL1/SLC7A5	5

**Table 3:** Molecular processes enrichment analysis.

Description	Gene Ratio	BgRatio	P Value	Adjusted P Value	Q Value	Gene ID	Count
ENZYME_BINDING	13/32	1949/15762	0.000	0.012	0.009	RB1/ELAVL1/LAMP2/ACSL3/DPP4/HIC1/MAP1LC3A/ATF4/HSPB1/RELA/GABARAPL1/MAPK3/LONP1	13
PROTEIN_DIMERIZATION_ACTIVITY	8/32	1033/15762	0.001	0.034	0.024	ELAVL1/ANO6/CYBB/IDH1/DPP4/ATF4/HSPB1/RELA	8
DNA_BINDING_TRANSCRIPTION_FACTOR_BINDING	5/32	376/15762	0.001	0.034	0.024	RB1/ATF4/HSPB1/RELA/GABARAPL1	5

**Table 4:** KEGG signal pathway enrichment analysis.

ID	Description	GeneRatio	BgRatio	pvalue	p.adjust	qvalue	geneID	Count
hsa04216	Ferroptosis	4/25	40/7914	6.56E-06	0.00110811	0.00067639	CYBB/ACSL4/ACSL3/MAP1LC3A	4
hsa03320	PPAR signaling pathway	4/25	76/7914	8.53E-05	0.00677765	0.00413709	SCP2/ACSL4/ACSL3/PLIN2	4
hsa04146	Peroxisome	4/25	83/7914	0.00012031	0.00677765	0.00413709	SCP2/ACSL4/ACSL3/IDH1	4
hsa01212	Fatty acid metabolism	3/25	57/7914	0.00072814	0.01671681	0.01020397	SCP2/ACSL4/ACSL3	3
hsa04140	Autophagy	4/25	137/7914	0.00081962	0.01671681	0.01020397	LAMP2/EIF2AK4/GABARAPL1/MAPK3	4
hsa04137	Mitophagy	3/25	65/7914	0.00106903	0.01671681	0.01020397	ATF4/RELA/GABARAPL1	3
hsa04920	Adipocytokine signaling pathway	3/25	69/7914	0.00127166	0.01671681	0.01020397	ACSL4/ACSL3/RELA	3
hsa05230	Central carbon metabolism in cancer	3/25	69/7914	0.00127166	0.01671681	0.01020397	IDH1/MAPK3/SLC7A5	3
hsa04934	Cushing syndrome	4/25	155/7914	0.00129914	0.01671681	0.01020397	RB1/FH/ATF4/MAPK3	4
hsa00061	Fatty acid biosynthesis	2/25	18/7914	0.00142115	0.01671681	0.01020397	ACSL4/ACSL3	2
hsa05161	Hepatitis B	4/25	162/7914	0.00152986	0.01671681	0.01020397	RB1/ATF4/RELA/MAPK3	4
hsa05212	Pancreatic cancer	3/25	75/7914	0.00161856	0.01671681	0.01020397	RB1/RELA/MAPK3	3
hsa05140	Leishmaniasis	3/25	76/7914	0.00168157	0.01671681	0.01020397	CYBB/RELA/MAPK3	3
hsa05220	Chronic myeloid leukemia	3/25	76/7914	0.00168157	0.01671681	0.01020397	RB1/RELA/MAPK3	3
hsa04621	NOD-like receptor signaling pathway	4/25	181/7914	0.00229848	0.02158016	0.01317257	CYBB/RELA/GABARAPL1/MAPK3	4
hsa05167	Kaposi sarcoma-associated herpesvirus infection	4/25	186/7914	0.00253831	0.02234137	0.01363721	RB1/RELA/GABARAPL1/MAPK3	4
hsa04211	Longevity regulating pathway	3/25	89/7914	0.00264395	0.02234137	0.01363721	ATF4/RELA/SESN2	3
hsa04657	IL-17 signaling pathway	3/25	93/7914	0.00299613	0.02411168	0.01471781	ELAVL1/RELA/MAPK3	3
hsa05203	Viral carcinogenesis	4/25	201/7914	0.00336092	0.02581798	0.01575934	RB1/ATF4/RELA/MAPK3	4
hsa04933	AGE-RAGE signaling pathway in diabetic complications	3/25	100/7914	0.00367949	0.02703625	0.01650297	CYBB/RELA/MAPK3	3
hsa00020	Citrate cycle (TCA cycle)	2/25	30/7914	0.00394786	0.02779951	0.01696887	IDH1/FH	2
hsa05166	Human T-cell leukemia virus 1 infection	4/25	219/7914	0.00456957	0.03047539	0.01860223	RB1/ATF4/RELA/MAPK3	4
hsa04066	HIF-1 signaling pathway	3/25	109/7914	0.00468852	0.03047539	0.01860223	CYBB/RELA/MAPK3	3
hsa05163	Human cytomegalovirus infection	4/25	225/7914	0.00503035	0.03053212	0.01863686	RB1/ATF4/RELA/MAPK3	4
hsa04668	TNF signaling pathway	3/25	112/7914	0.00505858	0.03053212	0.01863686	ATF4/RELA/MAPK3	3
hsa04722	Neurotrophin signaling pathway	3/25	119/7914	0.00598955	0.0349046	0.02130583	ATF4/RELA/MAPK3	3
hsa05219	Bladder cancer	2/25	41/7914	0.0072856	0.04082156	0.02491755	RB1/MAPK3	2
hsa04926	Relaxin signaling pathway	3/25	129/7914	0.00748798	0.04082156	0.02491755	ATF4/RELA/MAPK3	3
hsa00071	Fatty acid degradation	2/25	44/7914	0.00835662	0.04413339	0.0269391	ACSL4/ACSL3	2
hsa04210	Apoptosis	3/25	136/7914	0.00865822	0.04434061	0.02706558	ATF4/RELA/MAPK3	3

### 3.4 Protein–protein interaction network analysis of ferroptosis DEGs

**Figure 4:**
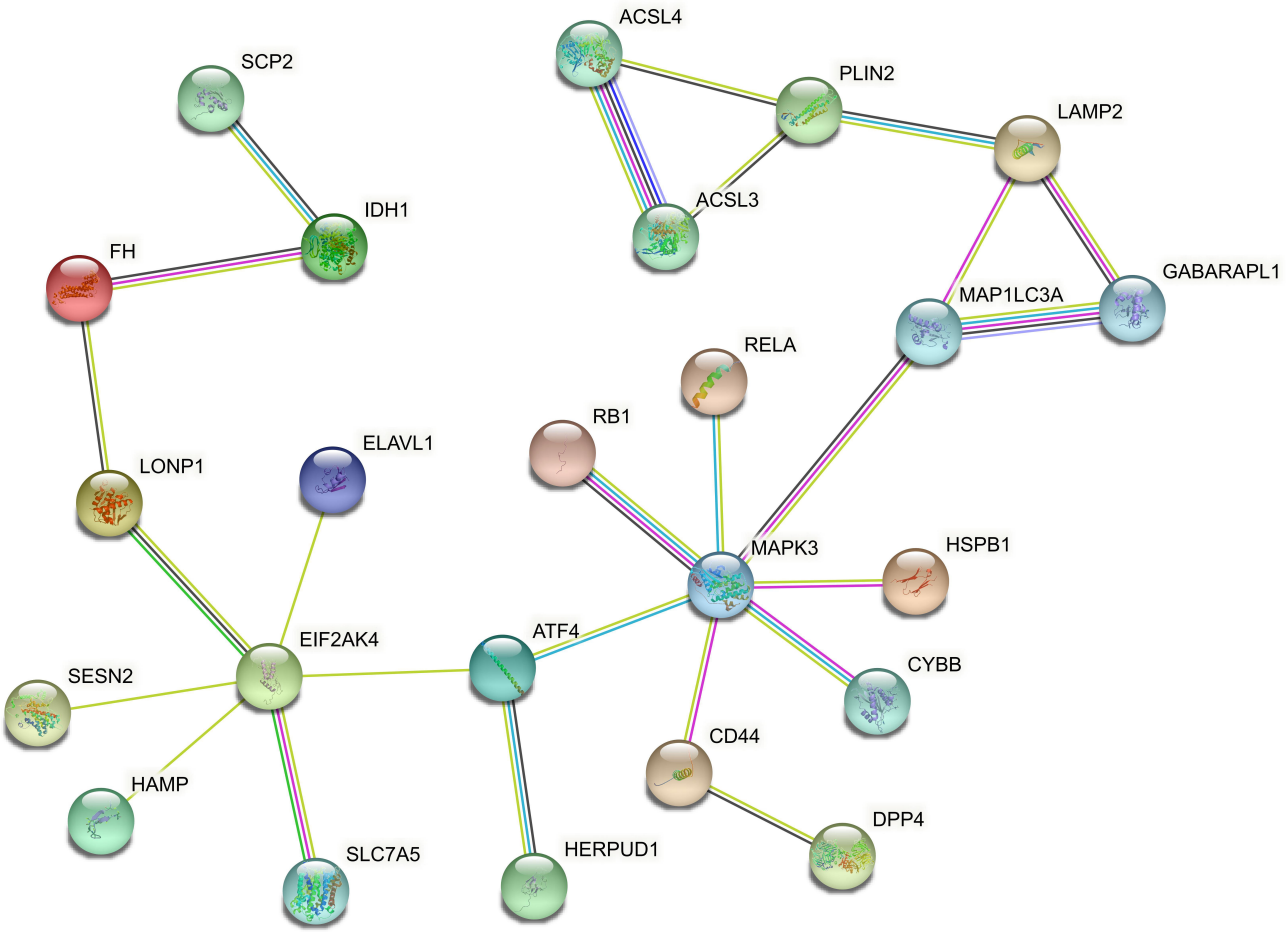
Protein–Protein Interaction Network Analysis of ferroptopsis DEGs: DCAF7, GABARAPL1, ACSL4, SESN2 and RB1 were found to be key elements in the PPI network.

In order to explore the association between the 33 differential ferroptosis genes, the protein interaction relationship among the 33 differential ferroptosis genes was analyzed by using the string database. The results predict that DCAF7, GABARAPL1, ACSL4, SESN2 and RB1 are key elements in the PPI network. The results are shown in **Figure 4**; **Table 5**.

**Table 5:** miRWalk prediction of MiRNA interaction.

mirnaid	refseqid	genesymbol	duplex	start	end	bindingp	energy	seed	accessibility	au	phylopstem	phylopflank	me	number_of_pairings	binding_region_length	longest_consecutive_pairings	position	validated	TargetScan	miRDB
hsa-miR-192-5p	NM_000321	RB1	CTGACCTATGAATTGACAGCC#GTTAGTTTTTAGGTCAA#.(((((((.((((((((....#)	4521	4538	1	-18.6	1	0.034781	0.647	0	0	-4.84971	15	17	8	3UTR	MIRT006435	1	1
hsa-miR-34a-5p	NM_001318509	ACSL4	TGGCAGTGTCTTAGCTGGTTGT#GCACCCCCAATCTGCCCCCAACTCCCAAAAGCTAGAACACTGCCAA#(((((((((.(((((((((((.#.)))))......)))))).))))))))).	4363	4409	1	-26.2	1	0.039272	0.544	0	0	-5.18008	20	29	9	3UTR	MIRT025215	1	1
hsa-miR-106b-5p	NM_001318509	ACSL4	TAAAGTGCTGACAGTGCAGAT#GTGCTGCAGGGCTTTAA#((((((.(((.((((((....#))))))))).)))))).	2930	2947	1	-19.7	0	0.001324	0.632	0	0	-8.30604	15	17	9	3UTR	MIRT175384	1	1
hsa-miR-34a-5p	NM_001318510	ACSL4	TGGCAGTGTCTTAGCTGGTTGT#GCACCCCCAATCTGCCCCCAACTCCCAAAAGCTAGAACACTGCCAA#(((((((((.(((((((((((.#.)))))......)))))).))))))))).	4072	4118	1	-26.2	1	0.039272	0.544	3.044349	2.677239	-5.18008	20	29	9	3UTR	MIRT025215	1	1
hsa-miR-106b-5p	XM_005262109	ACSL4	TAAAGTGCTGACAGTGCAGAT#GTGCTGCAGGGCTTTAA#((((((.(((.((((((....#))))))))).)))))).	2945	2962	1	-19.7	0	0.001324	0.632	0	0	-8.30604	15	17	9	3UTR	MIRT175384	1	1
hsa-miR-106b-5p	XM_006724635	ACSL4	TAAAGTGCTGACAGTGCAGAT#GTGCTGCAGGGCTTTAA#((((((.(((.((((((....#))))))))).)))))).	2563	2580	1	-19.7	0	0.001324	0.632	0	0	-8.30604	15	17	9	3UTR	MIRT175384	1	1
hsa-miR-106b-5p	XM_011530888	ACSL4	TAAAGTGCTGACAGTGCAGAT#GTGCTGCAGGGCTTTAA#((((((.(((.((((((....#))))))))).)))))).	2825	2842	1	-19.7	0	0.001324	0.632	0	0	-8.30604	15	17	9	3UTR	MIRT175384	1	1
hsa-miR-34a-5p	NM_022977	ACSL4	TGGCAGTGTCTTAGCTGGTTGT#GCACCCCCAATCTGCCCCCAACTCCCAAAAGCTAGAACACTGCCAA#(((((((((.(((((((((((.#.)))))......)))))).))))))))).	4381	4427	1	-26.2	1	0.039272	0.544	4.166855	4.296563	-5.18008	20	29	9	3UTR	MIRT025215	1	1
hsa-miR-106b-5p	NM_022977	ACSL4	TAAAGTGCTGACAGTGCAGAT#GTGCTGCAGGGCTTTAA#((((((.(((.((((((....#))))))))).)))))).	2948	2965	1	-19.7	0	0.001324	0.632	4.355351	4.601387	-8.30604	15	17	9	3UTR	MIRT175384	1	1
hsa-miR-34a-5p	NM_004458	ACSL4	TGGCAGTGTCTTAGCTGGTTGT#GCACCCCCAATCTGCCCCCAACTCCCAAAAGCTAGAACACTGCCAA#(((((((((.(((((((((((.#.)))))......)))))).))))))))).	4069	4115	1	-26.2	1	0.039272	0.544	3.633152	2.829761	-5.18008	20	29	9	3UTR	MIRT025215	1	1
hsa-miR-15b-5p	NM_031412	GABARAPL1	TAGCAGCACATCATGGTTTACA#CATGATGGCCAGCTGCTT#.((((((.(((((((.......#)))))))....)))))).	1576	1594	1	-17.4	1	0.000469	0.471	0	0	-6.34001	13	18	7	3UTR	MIRT247369	1	1
hsa-miR-6838-5p	NM_031412	GABARAPL1	AAGCAGCAGTGGCAAGACTCCT#AGGATTCTTGCTCCCATGCTGCTG#.(((((((..(((((((.((((#)))).)))))))....))))))).	1521	1545	1	-26.5	1	0.001299	0.5	0	0	-6.09222	18	24	7	3UTR	MIRT247380	1	1
hsa-miR-222-3p	NM_005828	DCAF7	AGCTACATCTGGCTACTGGGT#TCCAGTGGCCAGTATGTC#....((((((((((((((((.#)))))))))))).)))).	1312	1330	1	-25.2	0	0.001417	0.574	3.412256	2.046478	-9.54656	16	18	12	3UTR	MIRT046679	1	1
hsa-miR-222-3p	NM_005828	DCAF7	AGCTACATCTGGCTACTGGGT#ATCCACGGTCAGGTGTAGA#..((((((((((((..(((((#))))).)))))))))))).	6002	6021	1	-22.2	1	3.05E-05	0.529	1.202851	1.088908	-5.9255	17	19	12	3UTR	MIRT046679	1	1
hsa-miR-23c	NM_031459	SESN2	ATCACATTGCCAGTGATTACCC#GGGTCACAGCTGGTCTGTGTGT#..(((((.((((((....((((#))))....))))))..))))).	2259	2281	1	-20	0	5.05E-05	0.5	0	0	-8.23913	15	22	6	3UTR	MIRT067326	1	1

### 3.5 Prediction of miRNA interaction

**Figure 5:**
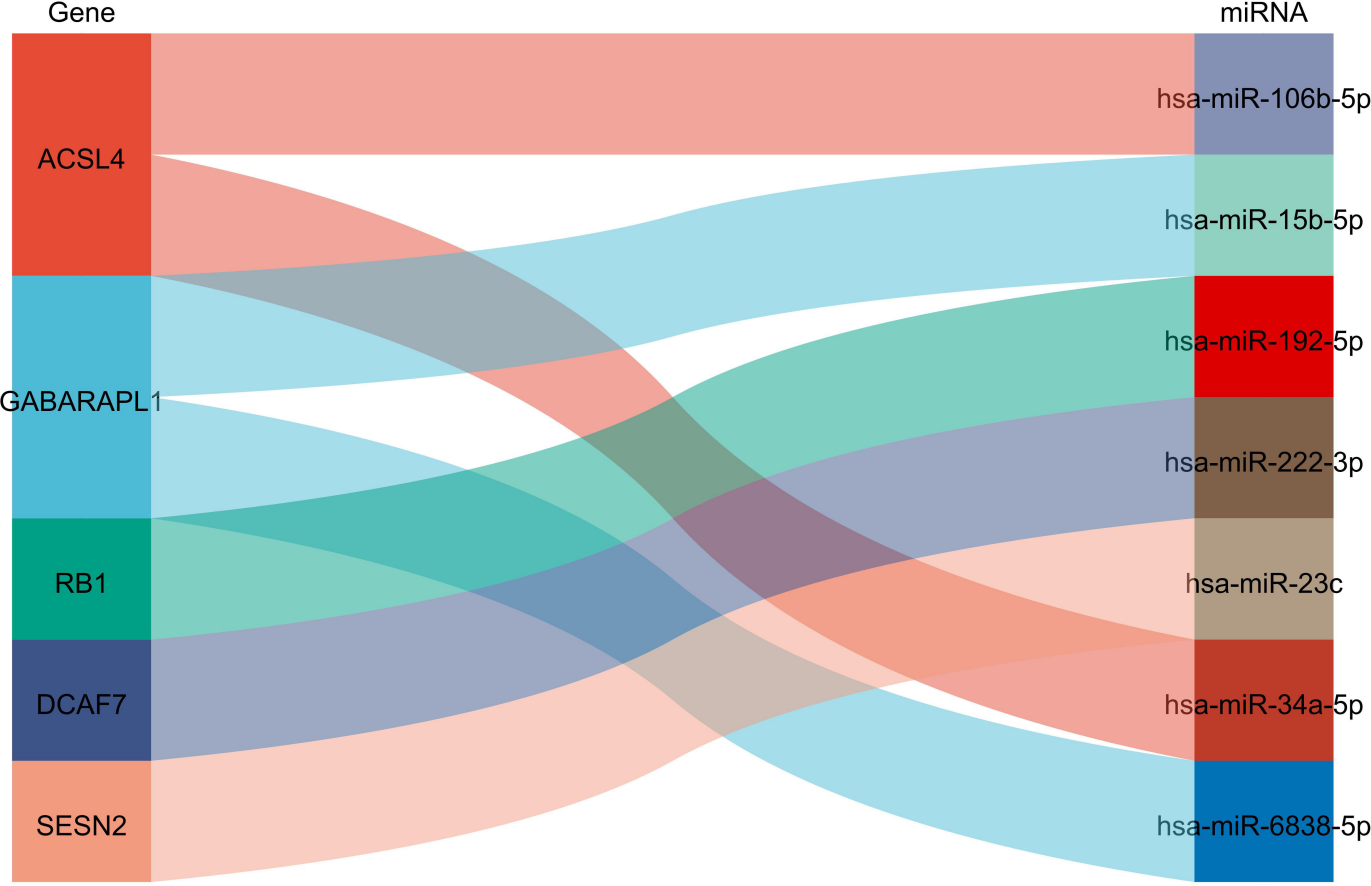
Prediction of miRNA interaction :ACSL4 could interact with miR108b-8p and miR34a-5p; GABARAPL1 could interact with mir15b-5p and miR-5838-5p, RB1 could interact with miR-192-5p, DCAF7 could interact with miR-222-3p while SESN2 could interact with miR-23c.

MiRNAs upstream of differential ferroptosis genes were obtained based on mirtarbase, and then the miRNA mRNA relationship pair network was constructed using Cytoscape, and analyzed using MiRwalk as shown in **Table 6**; **Figure 5**.

**Table 6:** Stromal score and immune score of the samples between the DM and DPN groups were estimated using the ESTIMATE algorithm.

	Group	Stromal Score	Immune Score	ESTIMATE Score
GSM2527022	DM	-634.39	3776.42	3142.03
GSM2527023	DM	-691.78	3902.09	3210.30
GSM2527024	DM	-777.02	3582.76	2805.74
GSM2527025	DM	-428.01	3737.13	3309.13
GSM2527026	DM	-582.34	3681.08	3098.74
GSM2527027	DM	-882.70	3716.42	2833.72
GSM2527034	DPN	-533.06	4061.98	3528.92
GSM2527035	DPN	-459.29	4024.72	3565.43
GSM2527036	DPN	-601.78	4061.10	3459.32
GSM2527037	DPN	-415.92	4162.82	3746.90
GSM2527038	DPN	-552.38	3950.22	3397.84
GSM2527039	DPN	-545.11	4129.07	3583.96

From **Figure 5**, it can be seen that ACSL4 can interact with miR108b-8p and miR34a-5p, GABARAPL1 interacts with mir15b-5p and miR-5838-5p, RB1 interacts with miR-192-5p, DCAF7 interacts with miR-222-3p, SESN2 interacts with miR-23c.

### 3.6 Immune microenvironment analysis

**Figure 6:**
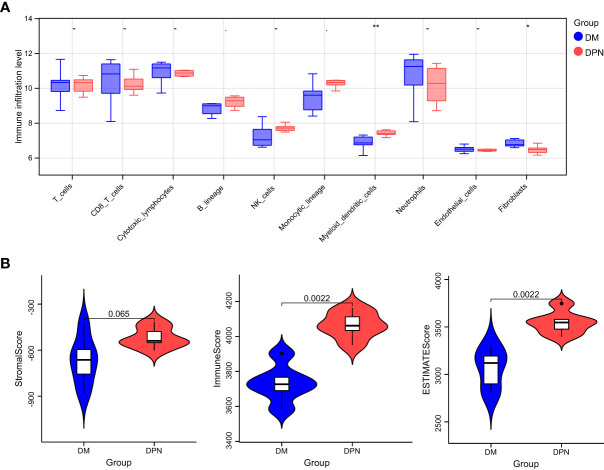
Immune microenvironment analysis. **(A)** Comparison of the various immune cell ratios between DPN and DM subjects. **(B)** Comparison of the immune score and matrix score in the infiltration level between the DPN and DM subjects.

Based on the expression profile data of GEO samples, the MCPcounter algorithm was used to calculate the immune cell types of each sample, and a total of 10 immune cell types were obtained. See attached **Table 4** for information. Then, we compared the difference of various immune cell ratios between DPN and DM. Taking P < 0.05 as the threshold, we obtained two kinds of differential immune cells (DICs) with significant differences: endothelial cells and fibroblasts. The comparison results between groups are shown in **Figure 6A**. The immune score and matrix score were calculated based on the estimate algorithm. The difference of immune score and matrix score in the infiltration level between DPN and DM was then analyzed. The results are shown in **Figure 6B** (1-3), **Table 6**.

## 4 Discussion

The complications associated with DM, such as DPN, have serious effects on the standard of life and socioeconomic welfare of patients (1). Henceforth, recent researches have been focusing on deciphering the underlying mechanism leading to the disease progression from DM to complication with DPN: In a study by Dewanjee et al., the implication of different molecular ad signaling pathways, oxidative stress, interleukins, nerve growth factor, and autophagy was thoroughly assessed in the investigation of the pathogenesis and progression of diabetic neuropathy (24). Along the same line of thought, we investigated the possible implication of ferroptosis in the pathogenesis and possible treatment of DPN. In this study, we identified the key genes involved in ferroptosis and further explored the mechanisms of ferroptosis in the pathogenesis of DPN. From the intersection of DEGs of the datasets GSE95849 and FerrDb, our study obtained 33 DEGs, including 19 downregulated genes (TFAP2C, HAMP, NNMT, MT3, HERPUD1, GRIA3, HIC1, MAP1LC3A, ATF4, HSPB1, RELA, BRD2, GABARAPL1, MAPK3, PLIN2, SLC7A5, YY1AP1, SESN2, LONP1) and 14 upregulated genes (DCAF7, RB1, ELAVL1, ANO6, SCP2, CYBB, CD44, LAMP2, ACSL4, ACSL3, IDH1, FH, EIF2AK4 and DPP4). Then, GO function and KEGG signal pathway enrichment analyses revealed that the 33 DEGs were involved in 27 biological processes, 10 cellular components, 3 molecular functions and 30 KEGG signal pathways. The genes were found to be mainly involved in response to extracellular stimulus and oxidative stress and related to ferroptosis signaling pathway. Further PPI network analysis predicted that DCAF7, GABARAPL1, ACSL4, SESN2 and RB1could be the key proteins while MirWalk predicted that miR108b-8p, miR34a-5p, mir15b-5p, miR-5838-5p, miR-192-5p, miR-222-3p and miR-23c could be mapped MiRNAs in the pathogenesis of DPN.

From 2012 till now, there have been only few studies that have highlighted the role of ferroptosis in the development of DPN: For instance, in a study by Feng et al., it was revealed that ferroptosis along the HIF-1α/HO-1 pathway lead to renal tubular injury and fibrosis in DM mice, partly also due to the increased production of ROS and accumulation of lipid peroxidation (13). In further studies related to the accumulation of lipid peroxidation revealed another key element, ACSL4, a key marker of ferroptosis and lipid peroxidation accumulation, which when down-regulated resulted in the inhibition of ferroptosis, finally improving the damage of renal tubular cells (25). Nevertheless, the role of ACSL4 in the mechanism of DPN has not been previously investigated. In a study by Cui et al, it was seen that ACSL4 promoted neuronal death *via* enhancing lipid peroxidation, identifying ACSL4 as a marker of ferroptosis, novel regulator of neuronal death and neuro-inflammation (26). Another marker of ferroptosis, SESN2 has been shown to play pivotal role in oxidative stress: In a study by Zhang et al., SESN2 was shown to influence oxidative stress of neurons by decreasing the expression of Nrf2 (27). Ferroptosis is characterized by the intracellular collection of lipid ROS, which are closely related and eventually result in the oxidation of lipids, thereby leading to cell membrane injury and cell death, which partly sustains the activity of ACSL4 and SESN2 (27, 28). Nevertheless, in this study, the bioinformatics analysis of the database revealed the possible involvement of 3 other targets which were not reported before: DCAF7, GABARAPL1 and RB1. After reviewing literature, we found that these genes have been related to ferroptosis in the mechanistic analysis of tumor development but they have not yet been mentioned in the context of DPN. These novel genes provide new concepts in the role ferroptosis in DPN.

When investigating the underlying pathways and mechanisms that could lead to the development of DPN in DM patients, we found that the differential genes were mainly involved in response to extracellular stimulus and oxidative stress mapped on the ferroptosis signaling pathway (13–16). The imbalance between the production of reactive oxygen species and their removal by antioxidant mechanisms over time can lead to tissue and organ damage (29). Henceforth we can speculate the possibility of ROS regulated ferroptosis in the mechanism behind the development of DPN.

Nevertheless, there is currently still a lack of understanding regarding potential biomarkers that can be used to specifically identify ferroptosis in clinical settings. Although proteins related to iron metabolism, such as ferritin, are elevated in the serum of patients with diabetes and related complications, their specificity cannot meet the requirements of clinical diagnosis. While investigating the immune-environment content of samples between DM and DPN patients, we found significant difference in the levels of endothelial cells and fibroblasts, which further speculates their possible involvement in the complication progression of DM. In a study by Li et al., it was found that exposure to hyperglycemic conditions induced excessive oxidative stress responses by fibroblasts and vascular endothelial cells, leading to lipid peroxidation and ferroptosis, a finding very similar to ours (30). In their study, Li et al. discovered that the inhibition of ferroptosis by Fer-1 reduced inflammation and promoted cell migration and proliferation, leading to accelerated wound healing.

The implication of ferroptosis in the pathogenesis of DPN is still work in progress. Henceforth, some of the limitations of our study are that by comparing established patient datasets, we obtained some theoretical targets marking the possible underlying mechanism defining the role of ferroptosis in DPN. Even though our results can provide insight for further *in vivo* and *in vitro* experiments to further decipher the role of ferroptosis in DPN, further validation and experiments are required to evaluate the findings of the study with further investigations about the therapeutic implications of the DEGs in the management of peripheral neuropathy in diabetic patients.

## 5 Conclusion

DPN is a serious complication in diabetic patient and the underlying mechanism is yet unclear. Ferroptosis has been recently intensively researched as a key process in the pathogenesis of diabetes. Henceforth, by means of bioinformatics analysis, 33 differential genes were first screened out. Functional pathway enrichment analysis revealed 127 significantly related biological processes, 10 cellular components, 3 molecular functions and 30 KEGG signal pathways. The biological processes that were significantly enriched were in response to extracellular stimulus. Key modules constructed by the protein–protein interaction network analysis and screening of genes related to the ferroptosis signaling pathway led to the confirmation of the following genes of interest: DCAF7, GABARAPL1, ACSL4, SESN2 and RB1. Further miRNA interaction prediction revealed the possible involvement of miRNAs such as miR108b-8p and miR34a-5p, mir15b-5p and miR-5838-5p, miR-192-5p, miR-222-3p, and miR-23c. While further investigating the immune-environment content of samples between DM and DPN patients, we found significant difference in the levels of endothelial cells and fibroblasts, which further speculates their possible involvement in the complication progression of DM.

## Data availability statement

The original contributions presented in the study are included in the article/**Supplementary Material**. Further inquiries can be directed to the corresponding author.

## Ethics statement

Ethical review and approval was not required for the study on human participants in accordance with the local legislation and institutional requirements. Written informed consent for participation was not required for this study in accordance with the national legislation and the institutional requirements.

## Author contributions

WZ is the corresponding author. WZ has designed the concept of the study and supervised the progress of the study. MT and YZ are co-first authors. MT and YC developed the concept and performed the numerical simulations. MT, FP and YC made the figures. MT, YC and AW wrote the paper. AW, HJ, RH and WZ supervised the project. All authors contributed to the article and approved the submitted version.
